# Standardizing and Improving Primary Care-Based Electronic Developmental Screening for Young Children in Federally Qualified Health Center Clinics

**DOI:** 10.1007/s10995-024-03970-y

**Published:** 2024-08-12

**Authors:** Gladys Felix, Alexis Deavenport-Saman, Sophia Stavros, Niloofar Farboodi, Ramon Durazo-Arvizu, Joanna Garcia, Larry Yin, Mona Patel Gera

**Affiliations:** 1 Division of General Pediatrics, Children’s Hospital of Los Angeles, 4650 Sunset Blvd, MS #76, Los Angeles, CA 90027 USA; 2 Keck School of Medicine of USC, Los Angeles, CA USA; 3 Biostatistics and Data Analysis Core, The Saban Research Institute, Children’s Hospital Los Angeles, Los Angeles, CA USA; 4 Institute of Health Equity, AltaMed Health Services Corporation, Los Angeles, CA USA

**Keywords:** Electronic developmental screening, Developmental delay, Autism, Children, Federally qualified health center

## Abstract

**Objectives:**

Many barriers to implementation of developmental screening in primary care exist, especially for children from under-resourced communities. Developmental screening is vital to early detection of developmental delay and autism spectrum disorder, and early intervention (EI) referral. This study sought to examine whether implementation of a standardized clinical workflow using electronic screening tools improved both rates of developmental screening, and the number of children identified at risk for developmental delay, in a federally qualified health center (FQHC).

**Methods:**

A retrospective study was conducted at an academic-affiliated FQHC. Electronic versions of the Ages and Stages Questionnaire 3 (ASQ-3) and Modified Checklist in Autism for Toddlers Revised (M-CHAT-R) were implemented at well-child visits. New clinical workflow training on developmental screening and EI referral was provided. Chi-square and Fisher’s Exact analyses were conducted.

**Results:**

ASQ-3 screening rates increased from 62.7 to 73.6% pre- to post-intervention. Post-intervention, there was a significant decrease in paper screens (*p* < .001), and a significant increase in the percentage of children with ASQ-3 results in the below cutoff range from 14.7 to 18.2% (*p* < .002). M-CHAT-R screening rates increased from 56.4 to 59.4% pre- to post-intervention. Post-intervention, there was a significant increase in electronic screens (*p* < .001).

**Conclusions for Practice:**

Implementation of electronic screening tools improved universal developmental screening in a FQHC. To decrease barriers in under-resourced communities, the use of electronic tools may decrease the rate of screening error seen with paper screening and have the potential to better identify children at risk for developmental delay.

## Significance

What is Already Known on this Subject?

What this Study Adds?

Developmental screening is critical to early detection of developmental delay and autism spectrum disorder, and referral to early intervention services; however, many barriers exist especially for children from under-resourced communities.

The primary care intervention in an FQHC increased universal developmental screening overall, as well as electronic screening and detection of potential developmental delay through training and uptake of screening practices at multiple clinic sites. Electronic screens increased for ASQ-3 and M-CHAT-R with subsequent decreases in paper-related screening errors.

Standardized developmental screening in the primary care setting, in addition to traditional developmental surveillance, is critical for early identification of children at-risk for developmental delay and improves referral to early intervention (EI) services (Lipkin et al., 2020b). In the U.S., it is estimated that 17% of children between the ages of 3–17 years have a developmental disability (Zablotsky et al., 2019). Without routine screening, at least 50% of children with developmental and behavioral diagnoses will remain unidentified by the time of kindergarten (Rice et al., 2014). EI before the age of 3, is vital to optimizing a child’s cognitive, language, motor, and socioemotional development, all of which contribute to school readiness and educational success. To improve early detection of developmental delay, the American Academy of Pediatrics (AAP) reinforced its policy recommendations to conduct standardized developmental screening at the 9-, 18-, and 30-month visits, and autism screening at the 18- and 24- month visits (Council on Children With Disabilities et al., 2006; Lipkin et al., 2020b).

Although AAP physician surveys report increasing developmental screening rates in the last decade of up to 63%, many challenges remain in implementation of screening programs. Clinicians continue to cite lack of staffing, time limitations, and lack of reimbursement as ongoing barriers to screening (Lipkin et al., 2020b; King et al., 2010; Sand et al., 2005). Results from the 2016 National Survey of Children’s Health revealed that less than one-third of children 9–35 months had received a developmental screen by a health care professional in the previous year (Hirai et al., 2018). Moreover, children had lower screening rates if they were racial/ethnic minorities, had less educated or lower-income parents, were from limited English proficiency (LEP) households and were publicly insured/uninsured; children from LEP households were also more likely to experience delays in both diagnosis and linkage to EI (Hirai et al., 2018; Zuckerman et al., 2017). In California, a report revealed that rates of developmental screening remain low for children in Medicaid at 25.4% (California Department of Health Care Services, 2022). Universal developmental screening in diverse and historically under-resourced patient populations is critical in improving this intervention service divide (Wallis et al., 2021).

Despite adopting an electronic health record (EHR), many practices continue to collect parent reported developmental screens on paper, contributing to poor screening rates. Paper screens can be lost, only partially filled in by parents, not fully scored by providers, or not scanned into the EHR for tracking. Provider documentation in the EHR is not always sufficient to determine if children with positive developmental screens were consistently referred to EI. To address these barriers to successful developmental screening, we created a standardized clinical workflow with an electronic developmental screening tool integrated into our EHR that automated and tracked scoring results. Training was provided to clinicians and staff in effective developmental screening and referral to EI services. Our clinical workflow adopted a clinic-wide approach in completing the electronic developmental tool, instead of relying on individual providers. We hypothesized that our workflow training intervention would strengthen the use of electronic over paper developmental screening. Electronic screen adoption in turn would lead to both an increased rate of developmental screening of children in a federally qualified health center clinics (FQHCs), and importantly identify more children at risk for developmental delay at an early age.

## Methods

### Study Design and Setting

A retrospective study was conducted across 5 clinic sites of a large community-based federally qualified health center (FQHC) in greater Los Angeles. The largest clinical site was co-located within a tertiary care teaching children’s hospital. About 80% of the pediatric patient population had Medicaid. We reviewed data over a three-year period, from 2014 to 2017 of children ages 9–24 months.

### Developmental Screeners and Inclusion / Exclusion Criteria

Children included in this study were those who had a well-child visit at the 9, 18, or 24-month age ranges between January 2014 to December 2017. To screen children for developmental delay and autism, we implemented the Ages and Stages Questionnaire-3 (ASQ-3) and the Modified Checklist for Autism in Toddlers, Revised (M-CHAT-R). The ASQ-3 is a validated parent questionnaire used for developmental screening of children ages 1 to 60 months across five developmental domains: communication, gross motor, fine motor, problem-solving, and personal-social skills (Squires et al., 2009; Olvera Astivia et al., 2017). Children included in the ASQ-3 analysis had a well-child visit at the 9, 18, or 24-month range. Children excluded were those with previously diagnosed developmental delay, or with history of prematurity. The excluded population did not require further screening or were more likely to have enhanced developmental surveillance and EI services. The M-CHAT-R is a validated parent questionnaire used to screen for autism in children ages 16 to 30 months (Robins, 2008; Robins et al., 2014; Beacham et al., 2018). Children included in the M-CHAT-R analysis had a well-child visit at the 18 or 24-month age range. Children excluded from the M-CHAT-R analysis were those with an existing autism diagnosis.

### Intervention

The two-part intervention included the integration of an electronic developmental screen in our EHR and the creation of a standardized clinical workflow to improve developmental screening and referral to EI services.

#### Electronic Screen Integration

To use an electronic version of the ASQ-3, a licensing agreement with the developer was required, included a yearly licensing fee and small cost per electronic screen used. The EHR company then built the electronic screen at a fixed cost. This was financially feasible through a state funded contract to support developmental screening. The M-CHAT-R was already available in the EHR and free of cost at the time of this intervention.

#### Clinical Workflow Creation

We partnered with developmental-behavioral pediatricians to assist in a series of live didactic training sessions of 56 clinicians (pediatricians, family medicine physicians, advanced practice practitioners), 75 pediatric residents, and 52 combined front office, medical assistant, and nursing staff (censed vocational nurses, and registered nurse supervisors) on developmental screening, referral to EI, and effective clinical workflow. The overarching structure of our workflow included the identification of an eligible child at the time of well child visit registration, provision of a tablet or computer to fill in the electronic screens, and the automation of the results in the EHR for clinician review prior to the visit starting. Electronic screens were provided in English and Spanish, and medical assistants were available to assist families as needed. At the time of the visit, the clinician used the electronic screen results to expedite developmental assessment and refer to EI services when appropriate. The workflow continued after the visit to include case management and closer follow up with the clinician to ensure linkage to EI services.

Two key elements to our workflow included customization to clinical site and iterative staff training through plan-do-study-act (PDSA) cycles. Our workflow was further divided by location and staff roles to individualize to specific clinic site needs. For example, in lower volume clinics the tablets were provided by front office staff to complete screens in the waiting room, while at higher volume clinics the tablets or computers were provided by a medical assistant to complete screens in patient rooms. The same training methods were implemented at all sites for fidelity and training quality was monitored through participant evaluations. Processes were reviewed on a quarterly basis and PDSA cycles were implemented to adapt and improve workflows at individual clinical sites.

### Pre–Post Intervention Analysis

We reviewed the rate and results of ASQ-3 and M-CHAT-R screening for a 15-month period pre-intervention, and 21-month period post-intervention.

#### Screening Rates

To examine changes in screening rates, we analyzed whether any screening with ASQ-3 or M-CHAT-R had taken place during the appropriate well-child visits, along with errors in screening: (1) paper screen completed, (2) electronic screen completed, (3) paper screen completed but not scanned into EHR, and (4) no screen done. Categories 3 and 4 reflected errors in our screening efforts.

#### Screening Results

To examine changes in the outcome of our screening, we then analyzed the number of children identified to be at risk for developmental delay or autism through screening. We reviewed ASQ-3 results across the 5 developmental domains assessed with 4 possible outcomes per domain: (1) Pass, (2) Borderline, (3) Below cutoff, and (4) Incomplete screen, for paper screens only partially filled in. Category 3 warranted referral to further developmental assessment, while category 4 reflected errors in screening results. When looking at the ASQ-3 results in aggregate, we categorized the overall result to below cutoff if at least one domain scored in that low range; borderline, if at least one domain scored in the middle range (without any below cutoff scores); and pass if all domains scored in the high range. M-CHAT-R results were similarly grouped into 4 categories using the scoring rubric including: (1) Low-risk, (2) Medium-risk, (3) High risk, and (4) Incomplete screens, for paper screens only partially filled in. Categories 2 and 3 warranted referrals to further developmental assessment, while category 4 reflected errors in screening results.

### Statistical Analyses

Stata 17 was used to conduct analyses. Descriptive statistics and frequencies were conducted. Two-sided chi-square tests were used to compare pre- and post -intervention screening rates for completed versus missed ASQ-3 and M-CHAT-R screens as well as pre- and post-intervention detection rates of children at risk for developmental delay. Chi-square tests were also conducted for pre- and post-intervention screening results of children at-risk for developmental delay identified through ASQ-3 screening across 5 developmental domains. A Bonferroni correction was conducted to adjust for multiple comparisons related to the study hypotheses. The reference *p*-value used was 5%/10 (*p* = .005) to determine a statistically significant result.

## Results

### Demographics

Overall, there were 9,988 children who were eligible for ASQ-3 screening. At 9 months of age, there were a total of 35% (3,493) eligible at 18 months of age there were 30% (2,992) eligible, and at 24 months there were 35% (3,503) eligible (Table 1). Pre- to post-intervention, there were significant differences by age, with an increase in those eligible for ASQ-3 screening at age 9 months, *p* < .001. There were 6,850 children who were eligible for M-CHAT-R screening, with, and 46% (3,133) eligible at 18 months and 54% (3,717) eligible at 24 months. There were no significant differences by age for the percent of children who were eligible for M-CHAT-R screening. Of the children eligible for both screenings, the majority (52%) were male. Over 47% of their families self-identified as White, and 41% as Hispanic/Latino; over 63% of families primarily spoke English and about 35% spoke Spanish.

**Table 1:** Demographics ASQ-3 and M-CHAT-R pre- and post-intervention

	Total	Pre-Intervention	Post-Intervention	*p*
	*n* (%) or Mean (SD)	*n* (%) or Mean (SD)	*n* (%) or Mean (SD)	
**ASQ Demographics**	(*n* = 9,988)	(*n* = 2,208)	(*n* = 4,065)	
Age				< .001
9 months	3493 (35)	696 (31.5)	2,797 (36.0)	
18 months	2992 (30)	662 (30.0)	2,330 (29.9)	
24 months	3503 (35)	850 (38.5)	2,653 (34.1)	
Sex				.783
Male	5526 (52.3)	1,161 (52.6)	4,065 (52.2)	
Female	4762 (47.7)	1,047 (47.4)	3,715 (47.8)	
Race/Ethnicity				< .001
White/Caucasian	4837 (48.4)	1,011 (45.8)	3,826 (49.2)	
Hispanic/Latino	4053 (40.6)	875 (39.6)	3,178 (40.8)	
Black/African American	448 (4.5)	154 (7.0)	294 (3.8)	
Asian	295 (3)	93 (4.2)	202 (2.6)	
Pacific Islander	46 (.5)	12 (0.5)	34 (0.4)	
Multi-racial	48 (.5)	12 (.5)	36 (0.5)	
Other/Unknown	261 (2.6)	51 (2.3)	210 (2.7)	
Primary language				.302
English	6351 (63.6)	1,414 (64.0)	4,937 (63.5)	
Spanish	3510 (35.1)	773 (35.0)	2,737 (35.2)	
Other	127 (1.3)	21 (1.0)	106 (1.4)	
**M-CHAT-R Demographics**	(*n* = 6,850)	(*n* = 1,520)	(*n* = 5,330)	
Age				.999
18 months	3133 (45.7)	667 (43.9)	2,466 (46.3)	
24 months	3717 (54.3)	853 (56.1)	2,864 (53.7)	
Sex				.971
Male	3625 (52.9)	805 (53.0)	2,820 (52.9)	
Female	3225 (47.1)	715 (47.1)	2,510 (47.1)	
Race/Ethnicity				< .001
White/Caucasian	3229 (47.1)	701 (46.1)	2,528 (47.4)	
Hispanic/Latino	2841 (41.5)	582 (38.3)	2,259 (42.4)	
Black/African	318 (4.6)	108 (7.1)	210 (3.9)	
American				
Asian	220 (3.2)	76 (5.0)	144 (2.7)	
Pacific Islander	34 (.5)	8 (0.5)	26 (0.5)	
Multi-racial	35 (.5)	5 (0.3)	30 (0.6)	
Other/Unknown/American Indian	173 (2.5)	40 (2.6)	133 (2.5)	
Primary language				.117
English	4298 (63.1)	961 (63.3)	3,337 (63.1)	
Spanish	2471 (36.3)	544 (35.8)	1,927 (36.4)	
Other	39 (.6)	14 (0.9)	25 (.5)	

### Screening Rates for Completed Electronic and Paper Screens Compared to Missed ASQ-3 and M-CHAT-R Screens

The total screening rate for paper and electronic ASQ-3 screens was 71.2% (Table 2). There was a significant difference in the rate of screening from pre- to post-intervention. Pre-intervention, ASQ-3 screening rate was 62.7% through paper screening. Pre- to post-intervention, there was a decrease in paper screens, *p* < .001, while electronic screens increased from 0 to 30%. The ASQ-3 screening rate increased to 73.6%, comprised of 43.9% paper screens and 29.7% electronic screens, and the rate of missed screens decreased, *p* < .001. The total rate for paper and electronic M-CHAT-R screens was 58.2%. Pre-intervention, M-CHAT-R screening rate was 56.4%, with 40.8% paper and 15.6% electronic screens. Pre- to post-intervention, there was a significant decrease in paper screens compared to electronic screens, *p* < .001. The M-CHAT-R screening rate increased to 59.4%, comprised of 23.9% paper screens and 35.5% electronic screens, and a lower rate of missed screens, *p* < .001.

**Table 2:** Screening rates for completed electronic and paper compared to missed ASQ-3 and M-CHAT-R screens pre- and post-intervention

	Total Screening Rate	Pre-intervention Screening Rate	Post-intervention Screening Rate	*p*
	*n* (%)	*n* (%)	*n* (%)	
**ASQ-3 Screening**	(*n* = 9,972)	(*n* = 2,192)	(*n* = 7,780)	
ASQ Screens				< .001
Paper screens	4,787 (48.0)	1,375 (62.7)	3,412 (43.90)	
Electronic screens	2,314 (23.2)	0	2,314 (29.70)	
Completed, but missing paper screens in EMR (missed screens)	228 (2.3)	106 (4.8)	122 (1.6)	
No ASQ completed (missed screens)	2,643 (26.50)	711 (32.4)	1,932 (24.8)	
**MCHAT-R Screening**	(*n* = 6,792)	(*n* = 1,462)	(*n* = 5,330)	
M-CHAT-R Screens				
Paper screens	1,870 (27.5)	597 (40.8)	1,273 (23.9)	< .001
Electronic screens	2,120 (31.2)	228 (15.6)	1,892 (35.5)	
Completed, but missing paper screens in EMR (missed screens)	67 (1.0)	27 (1.8)	40 (0.8)	
No MCHAT completed (missed screens)	2,735 (40.3)	610 (41.7)	2,125 (39.9)	

^a^Adjusted *p*-value: *p* < .005

Note: For ASQ-3s, data were missing for 16 children

### Screening Results for ASQ-3 and M-CHAT-R: Identifying Children at Risk for Developmental Delay

Overall, for ASQ-3 outcomes, the rate of potential developmental delay (borderline and below cutoff) was 37% (Table 3). There were limited errors in incomplete screens. Pre- to post-intervention, there was a significant increase in the rate of ASQ-3s identifying a below cutoff result (14.7–18.2%), *p* < .002. There was a corresponding decrease in results with a pass result (72.6–58.1%) and missed screens, *p* < .001. Overall, for M-CHAT-R outcomes, the rate of children identified as at risk for autism, or having medium- or high-risk scores, was 9.1%. There were also fewer errors in incomplete screens. There was an increase in the rate of M-CHAT-Rs with a low-risk result from 85.1 to 90.2% and decreases in medium- and high-risk results, *p* < .001. There was a significant decrease in paper screens compared to electronic screens, *p* < .001.

**Table 3:** Screening results for ASQ-3 and MCHAT-R: identifying children at risk for developmental delay pre- and post-intervention

	Total ASQ Results	Pre-intervention ASQ Results/Scores	Post-intervention ASQ Results/Scores	*p*
	*n* (%)	*n* (%)	*n* (%)	
**ASQ-3 Screening Results**	(*n* = 7,101)	(*n* = 1,375)	(*n* = 5,726)	
ASQ- 3 Results				< .001
Pass	4,323 (60.9)	998 (72.6)	3,325 (58.1)	
Borderline	1,378 (19.4)	143 (10.4)	1,235 (21.6)	
Below Cutoff	1,248 (17.6)	202 (14.7)	1,046 (18.2)	
Paper Incomplete Screens	152 (2.1)	32 (2.3)	120 (2.1)	
**MCHAT-R Screening** **Results**	(*n* = 3,952)	(*n* = 824)	(*n* = 3,128)	
M-CHAT-R Results				< .001
Low-risk	3,524 (89.2)	701 (85.1)	2,823 (90.2)	
Medium-risk	239 (6.0)	60 (7.3)	179 (5.7)	
High-risk	124 (3.1)	32 (3.9)	92 (2.9)	
Paper Incomplete Screens	65 (1.6)	31 (3.8)	34 (1.1)	

^a^Adjusted *p*-value: *p* < .005

Note. Data for 38 were missing for M-CHAT-Rs

### ASQ-3 Screening Results: Children at Risk for Delay by Type of Developmental Domain

There were significant differences from pre- to post-intervention of the ASQ-3 results for identifying risk of communication delay, *p* < .001, gross motor delay, *p* < .001, fine motor delay, *p* < .001, problem solving delay, *p* < .001, and personal social delay, *p* < .002 (Table 4). For all outcomes except personal social delay, the percent of screeners completed with below cutoff results increased.

**Table 4:** ASQ-3 results of children at risk for developmental delay across five domains of development pre- post-intervention

Domains of Development	Pre-intervention ASQ-3 Domain Results	Post-intervention ASQ-3 Domain Results	*p*
	*n* (%)	*n* (%)	
	(*n* = 1,375)	(*n* = 5,726)	
Communication			<.001
Pass	1197 (87.1)	4547 (79.4)	
Borderline	95 (6.9)	687 (12)	
Below Cutoff	83 (6)	492 (8.6)	
Gross Motor			<.001
Pass	1206 (87.7)	4765 (83.2)	
Borderline	78 (5.7)	531 (9.3)	
Below Cutoff	91 (6.6)	430 (7.5)	
Fine Motor			<.001
Pass	1223 (88.9)	4903 (85.6)	
Borderline	57 (4.1)	411 (7.2)	
Below Cutoff	95 (6.9)	412 (7.2)	
Problem Solving			<.001
Pass	1226 (89.2)	4864 (84.9)	
Borderline	47 (3.4)	432 (7.5)	
Below Cutoff	102 (7.4)	430 (7.5)	
Personal Social			.002
Pass	1215 (88.4)	4953 (86.5)	
Borderline	69 (5)	438 (7.6)	
Below Cutoff	91 (6.6)	335 (5.9)	

^a^Adjusted *p*-value: *p* < .005

## Discussion

Our intervention produced a sustained increase in developmental screening across a diverse population of nearly 10,000 well-child visits at a community based FQHC. The overall ASQ-3 screening rate increased from 63 to 72%, and of those post-intervention screens, 30% were completed using the new electronic ASQ-3 screen. The M-CHAT-R screening rate increased from 56 to 59%, but there was a large improvement in completed screens via use of the previously available electronic M-CHAT-R screen from 15 to 35%. Continued education and training of our staff through PDSA cycles was important in maintaining a sustained increase in screening over the 2-year post-intervention study period. Training had to be tailored to each clinical site’s needs, included the training of front office staff, medical assistants, nurses, pediatricians, advanced practice practitioners, and family medicine physicians, and occurred on a regular basis to provide screening rate feedback, troubleshoot barriers, and account for inherent turnover in medical staff and resident learners.

We achieved partial adoption of electronic screening, and our study suggests that increased implementation of both paper and electronic developmental screening identified more children at risk for developmental delay within our under-resourced population. We exceeded the national developmental screening target rates of 36% set by Healthy People 2030 (ODPHP, (n.d.) A recent study in community clinics had similar autism screening rates as our study (43.4-52.4%) after implementing automatic reminders in the EHR (Campbell et al., 2021). Another study found that personalized notifications to physicians from an automated electronic screening tool increased screening rates (Carroll et al., 2014). Pre-screening prior to well child visits using electronic or web-based frameworks offers yet another promising model for FQHCs and are modifiable to site-specific practices (Coker et al., 2014). Furthermore, improving data infrastructure, administrative coordination, and better integrating screening with comprehensive services may help to increase access to services for some families (Horm et al., 2024).

When examining the children screened with an ASQ-3, children identified with potential delay increased from 14% pre-intervention to 18% post-intervention, indicating a need for referral to full developmental assessment and EI services. In addition, the children with borderline scores on the ASQ-3 also substantially increased the number of children needing enhanced developmental monitoring beyond the AAP Well-Child Visit schedule (Hagan et al., 2017). This increase in identified risk was seen across 4 of the 5 developmental domains assessed, including communication, gross motor, fine motor, and problem solving. Our intervention resulted in an overall increase in identification of children at risk for developmental delay.

In contrast to developmental screening, however, improvement in autism screening resulted in a small decrease in both medium and high-risk scores, from 7.3 to 5.7%, and 3.9–2.9% respectively. We suspect this may be the result of improved universal screening with our intervention, moving away from targeted use of screens when there is a parent or provider concern, and reflecting a more accurate rate of autism risk in our population.

In addition to higher screening rates, we also observed an improvement in screening errors with the adoption of electronic screens. Both the number of incomplete screens and paper screens lost, improved for both ASQ-3 and M-CHAT-R initiatives. Losing a paper screen prior to scanning into our EHR limits our ability to systematically track and reach out to children with positive screens outside of the clinical encounter. Increasing training for both primary care physicians and staff can help to promote screening implementation and decrease screening errors (Bellesheim et al., 2020; Mazurek et al., 2021). Improved clinical workflows around electronic developmental screens may decrease errors, and further allow for the building of robust referral and linkage systems to EI to ultimately improve child outcomes.

We identified several barriers to adopting electronic screens. Wireless internet infrastructure and physical space constraints have a large impact on the collection of electronic data. Inadequate clinic system wireless connectivity impacted the use of tablets in the waiting room. We pivoted to the use of wired computer workstations in the patient exam room in a protected patient-mode. This operational workflow change was further impacted by timely room availability and gave parents less time to fill in their electronic screens. The lack of sufficient exam rooms and computers often required our staff to revert to paper screens to ensure screening was completed. The flexibility between electronic and paper screening during this transition period to a new clinical workflow was vital for provider and staff to maintain clinic efficiency. Survey fatigue may have impacted rates of M-CHAT-R completion, as it was the last survey requested from families in addition to other clinical forms required at the visit. Since developmental screening occurred later in the visit, this may have ultimately impacted screen completion rates, adequate time for physician review, and appropriate referral to EI in our busy clinics. Despite barriers, we implemented screening in a large sample conducted in FQHC clinics. Another study implemented developmental screening in primary care clinics, assessing screening in over 2,000 well-child visits in 39% of Latinos, whereas in the present study screening was assessed in nearly 10,000 well-child visits in over 40% of Latinos (Zuckerman et al., 2021).

### Clinical and Policy Implications

The positive impact of developmental screening for young children is widely accepted and our study shows it is feasible to improve screening rates in a limited resource setting. When selecting a developmental screener, it is important to consider cost of implementation and local insurance reimbursement models. In addition to the ASQ-3, there are AAP-endorsed validated screening tools with differing cost profiles, such as the Survey of Well-Being for Young Children (SWYC) and the Parents’ Evaluation of Developmental Status (PEDS), (Sheldrick et al., 2019, 2020; Lipkin et al., 2020a, b), some of which are already integrated in popular EHR systems without added costs. EHR integration allows for improved billing and reimbursement of screening, which can sustain developmental screening programs, support staff and ongoing PDSA cycles, and fund programs that help link families to needed EI resources. In California specifically, passage of legislation in 2020 allowed providers to bill Medicaid for developmental screening, further incentivizing this practice during well-child visits. Legislation nationwide may further promote adoption of screening programs and minimize concern for cost as a barrier.

This study had various limitations. Findings may not be generalizable to non-FQHC settings. As a cross-sectional study, it was not possible to isolate the impact on screening rates of an electronic tool from those of other factors, such as staff training and attention. During the study period, developmental screening was not routinely conducted at 30 months, which is currently recommended, instead of 24 months (Lipkin et al., 2020a, b). Study results may have potentially been different if the M-CHAT-R and the ASQ-3 were conducted at the 24- and 30- month visits. It was also not possible to confirm the developmental outcomes of the children, as this was out of the scope of this study. Lastly, the errors in the completed, but missing paper screens in EHR or incomplete ASQ-3 and M-CHAT-R screens limited accurate rates of risk for developmental delay. Conversely, our study had many strengths, namely the large sample size allowing for more power to detect effects and sampling of a racially and ethnically diverse population. We suspect that Hispanic/Latino children may have been undercounted as data may not have been reported accurately; it is possible that White race was selected without adding Hispanic/Latino ethnicity when applicable, as the Hispanic/Latino population served by our FQHC is higher than reported in the study. Finally, there are few studies looking at developmental screening programs in under-resourced communities with high percentage of Hispanic/Latino children and families who speak Spanish as their primary language.

## Conclusion

Implementation of electronic developmental screening, together with iterative clinic-wide training, can improve rates of developmental screening of young children FQHCs. Increased screening may detect larger numbers of children at-risk for developmental delay, which is crucial to improving developmental outcomes of children, especially in diverse, under-resourced, and multi-lingual communities. In addition, further studies are needed to explore the impact of integrating electronic screens into EHRs on linking children to EI services beyond the clinical encounter, monitoring population risk and developmental outcomes, and allowing for consistent reimbursement from insurance providers to achieve a sustainable screening program.

## Data Availability

Available upon request.

## Code Availability

Available upon request.
