# Correction: Standardizing and Improving Primary Care-Based Electronic Developmental Screening for Young Children in Federally Qualified Health Center Clinics

**DOI:** 10.1007/s10995-024-04031-0

**Published:** 2024-11-18

**Authors:** Gladys Felix, Alexis Deavenport-Saman, Sophia Stavros, Niloofar Farboodi, Ramon Durazo-Arvizu, Joanna Garcia, Larry Yin, Mona Patel Gera

**Affiliations:** 1 Division of General Pediatrics, Children’s Hospital of Los Angeles, 4650 Sunset Blvd, MS #76, Los Angeles, CA 90027 USA; 2 Keck School of Medicine of USC, Los Angeles, CA USA; 3 Biostatistics and Data Analysis Core, The Saban Research Institute, Children’s Hospital Los Angeles, Los Angeles, CA USA; 4 Institute of Health Equity, AltaMed Health Services Corporation, Los Angeles, CA USA

**Correction to: Maternal and Child Health Journal (2024) 28:1716–1725**

10.1007/s10995-024-03970-y

The original version of this article unfortunately contained a typo in fifth co-author name.

The given name and family name of author Ramon Durazo-Arvizu were swapped. Now, the correct author names are presented in this correction article.

The original article has been corrected.

